# Effects of Visfatin on Intracellular Mechanics and Catabolism in Human Primary Chondrocytes through Glycogen Synthase Kinase 3β Inactivation

**DOI:** 10.3390/ijms22158107

**Published:** 2021-07-28

**Authors:** Shun-Fu Chang, Kuo-Chin Huang, Kuan-Han Lee, Yao-Chang Chiang, Wei-Ru Lee, Rong-Ze Hsieh, Yu-Ping Su, Shun-Chi Wu

**Affiliations:** 1 Department of Medical Research and Development, Chiayi Chang Gung Memorial Hospital, Chiayi 613, Taiwan; sfchang@cgmh.org.tw (S.-F.C.); atmw113106@gmail.com (R.-Z.H.); 2 Department of Orthopaedics, Chiayi Chang Gung Memorial Hospital, Chiayi 613, Taiwan; kc2672@adm.cgmh.org.tw; 3 College of Medicine, Chang Gung University, Taoyuan 333, Taiwan; 4 Department of Pharmacy, Chia Nan University of Pharmacy and Science, Tainan 717, Taiwan; kuanhanlee@mail.cnu.edu.tw; 5 Chronic Diseases and Health Promotion Research Center, Division of Basic Medical Sciences, Department of Nursing, Chang Gung University of Science and Technology, Chiayi 613, Taiwan; yaochang.chiang@gmail.com; 6 Department of Engineering and System Science, National Tsing Hua University, Hsinchu 300, Taiwan; rainpeoplerainpeople@gmail.com; 7 Graduate Institute of Clinical Medical Sciences, College of Medicine, Chang Gung University, Taoyuan 333, Taiwan; 8 Department of Orthopaedics and Traumatology, Veterans General Hospital, Taipei 112, Taiwan; 9 Department of Surgery, School of Medicine, National Yang-Ming University, Taipei 112, Taiwan

**Keywords:** elasticity, glycogen synthase kinase 3β, intracellular mechanics, osteoarthritis, primary human chondrocytes, viscosity, visfatin

## Abstract

Osteoarthritis (OA) is still a recalcitrant musculoskeletal disease on account of its complex biochemistry and mechanical stimulations. Apart from stimulation by external mechanical forces, the regulation of intracellular mechanics in chondrocytes has also been linked to OA development. Recently, visfatin has received significant attention because of the clinical finding of the positive correlation between its serum/synovial level and OA progression. However, the precise mechanism involved is still unclear. This study determined the effect of visfatin on intracellular mechanics and catabolism in human primary chondrocytes isolated from patients. The intracellular stiffness of chondrocytes was analyzed by the particle-tracking microrheology method. It was shown that visfatin damages the microtubule and microfilament networks to influence intracellular mechanics to decrease the intracellular elasticity and viscosity via glycogen synthase kinase 3β (GSK3β) inactivation induced by p38 signaling. Further, microtubule network destruction in human primary chondrocytes is predominantly responsible for the catabolic effect of visfatin on the cyclooxygenase 2 upregulation. The present study shows a more comprehensive interpretation of OA development induced by visfatin through biochemical and biophysical perspectives. Finally, the role of GSK3β inactivation, and subsequent regulation of intracellular mechanics, might be considered as theranostic targets for future drug development for OA.

## 1. Introduction

Osteoarthritis (OA) is the most common articular cartilage degeneration disease. People suffering from OA may experience a poor lifestyle because of the painfulness, disability, and debilitation associated with the condition [1,2]. However, OA is still a recalcitrant disease, as much of the mechanisms of the complex biochemical pathways and mechanical stimulation involved in the onset of this condition remain to be elucidated. The chondrocyte is the only type of cell in articular cartilage. The homeostasis of chondrocyte dynamics plays a critical role in the maintenance of cell physiology and tissue architecture. Upon the onset of OA, an imbalanced metabolism in chondrocytes gradually results in a higher secretion of catabolic factors, e.g., matrix metalloprotease (MMP) and cyclooxygenase 2 (COX2), than those of the anabolic factors, e.g., collagen II and aggrecan. This means that the degradation rate in cartilage becomes gradually higher than the regeneration and repair rate, which finally leads to cartilage destruction [1,2]. COX2 is the major enzyme in prostaglandin (PG) synthesis. PG upregulation in cartilage has been positively correlated with inflammation and disease development, including OA [3]. Hence, increase of COX2 expression/activity has been indicated as a catabolic event in chondrocytes [3].

Currently, obesity is still the most influential risk factor for OA. In addition to the mechanical overloading effect, the secretion of adipokines (including chemerin, leptin, resistin, and visfatin) from white adipose tissue and synovial tissue in obese subjects has been recognized as the major sources of chronic inflammation initiating cartilage damage [4,5,6,7]. Among these adipokines, recent research has been focused on visfatin because of accumulating in vitro data showing its role in inducing inflammatory response (interleukin-6, tumor necrosis factor-alpha, and nuclear factor kappa B), degradative effect (oxidative stress and apoptosis), and imbalanced metabolism (MMPs upregulation and collagen II/aggrecan deregulation) in chondrocytes [6,7,8]. Moreover, clinical evidence has found a positive correlation between the serum/synovial visfatin level and OA progression in both obese and nonobese patients [6,7,8,9,10,11]. Visfatin is a pleiotropic protein, which is responsible for both physiological and pathophysiological processes. Its intracellular form, called nicotinamide phosphoribosyltransferase (NAMPT), possesses enzyme activity to catalyze nicotinamide mononucleotide formation, which benefits physiological essential metabolism. Its secretory extracellular form, visfatin, usually acts as an inflammatory cytokine, which attracts much more attention because of its pathophysiological role in the development of different types of diseases, including OA [6,7,8,9,10,11]. The pharmacological intervention of visfatin inhibitors in different diseases, including diabetes and cancer, has been examined [12]. Recently, it has been proposed that visfatin might also serve as a potential clinical theranostic target in OA diagnosis and treatment [12], but its precise mechanisms have still not been elucidated clearly and adequately. Thus, more comprehensive investigations through different methods, e.g., biomechanics, are still necessary.

In order to support and maintain normal motion, articular cartilage must temporally and spatially expose to various mechanical environments. Here, cartilage must absorb and alleviate mechanical pressure and then adapt to these mechanical stimuli quickly [13,14]. In addition to these extracellular mechanical modulations, data have suggested that the intracellular biophysical behavior of chondrocytes might also be vital for cartilage health and degeneration pathogenesis [15,16,17]. It has been shown that the intracellular mechanics of chondrocytes isolated from normal cartilage and OA cartilage are different [16,18,19]. These differences might result in a stiffer or softer cytoplasmic environment in chondrocytes according to various stimuli. The cytoskeleton has been recognized as vital in the maintenance and modulation of cell morphology. The dynamic deformations of the cells responding to various stimuli are mainly controlled by the three-dimensional stress fiber networks formed by the cytoskeletal proteins, i.e., actin (microfilaments), tubulin (microtubules), and vimentin (intermediate filaments) [20]. Hence, the architecture and polymerization status of the cytoskeleton have been directly associated with the intracellular mechanics changes of cells [17,18,19]. In the cartilage, the cytoskeleton has been indicated to serve as a mechanical communicator between the extracellular matrix and chondrocytes [21]. This means that the cytoskeleton directly responds to exogenous stimuli and subsequently initiates and regulates the mechanotransduction to influence cell function. Most previous studies exploring the intracellular mechanics of chondrocytes have only observed the correlations between intracellular mechanical changes and OA progression [16,22,23,24]. However, the underlying molecular mechanisms have not been adequately elucidated.

In the present study, the effect of visfatin on intracellular mechanics and catabolism in human primary chondrocytes, and their underlying mechanisms, were examined. The intracellular mechanics of chondrocytes was analyzed by the particle-tracking microrheology (PTM) method, which is a technique to examine the rheological (viscoelasticity) properties of materials, including that of cells [25]. By observing the motion of tracer particles embedded therein, PTM facilitates the viscoelasticity of materials to be inferred based on the generalized Stokes-Einstein relation [25]. Our results found that visfatin damages the microtubule and microfilament networks to affect intracellular mechanics, which decreases intracellular elasticity and viscosity properties in human primary chondrocytes through glycogen synthase kinase 3β (GSK3β) inactivation induced by p38 signaling; also, the microtubule network destruction in human primary chondrocytes is predominantly responsible for the visfatin catabolic effect on COX2 upregulation. Our study comprehensively elucidated the visfatin catabolic effect on human primary chondrocytes through biochemical and biophysical regulation, and proposes that the roles of GSK3β inactivation and subsequent intracellular changes in mechanics might be considered as potential theranostic targets for future drug development for OA. 

## 2. Results

### 2.1. Visfatin Inactivates GSK3β to Increase Catabolic COX2 Expression in Human Primary Chondrocytes

**Figure 1:**
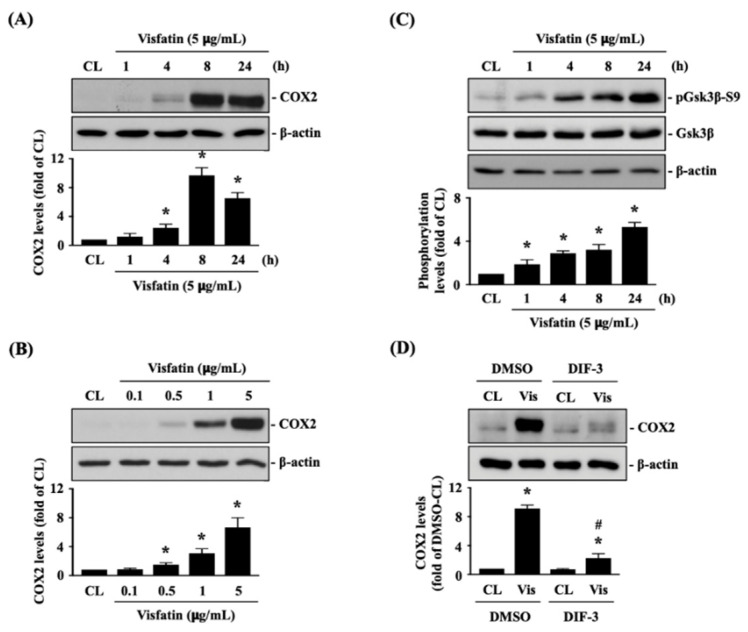
Visfatin inactivates GSK3β to increase catabolic COX2 expression in human primary chondrocytes. (**A**–**C**) Human primary chondrocytes were kept as controls (CL) or treated with 5 μg/mL of visfatin for 1, 4, 8 and 24 h or treated with 0.1, 0.5, 1 or 5 μg/mL of visfatin for 8 h and then (**A**,**B**) COX2 protein expression and (**C**) GSK3β-Ser-9 phosphorylation were analyzed by Western blot. (**D**) Human primary chondrocytes were pretreated with DMSO or DIF-3, a GSK3β activator, for 1 h and then kept as controls (CL) or treated with 5 μg/mL of visfatin (Vis) for 8 h. COX2 expression in human primary chondrocytes was analyzed by Western blot. Results in (**A**–**D**) are representative of three independent experiments with similar results. Statistical data in (**A**–**D**) are mean ± SEM from three independent experiments. *, *p* < 0.05 vs. untreated control cells (CL) or DMSO-CL-treated cells. #, *p* < 0.05 vs. DMSO-visfatin (Vis)-stimulated cells.

Human primary chondrocytes were kept as controls or treated with 5 μg/mL of visfatin for 1, 4, 8 and 24 h or treated with 0.1, 0.5, 1 or 5 μg/mL of visfatin for 8 h, and then COX2 protein (catabolic marker) expression was analyzed. Treatment of human primary chondrocytes with visfatin induces COX2 protein expression in a time and dose-dependent manner (Figure 1A,B). Visfatin (5 μg/mL) induced a rapid increase in COX2 within 4 h, which reached a peak level approximately nine times that of untreated controls after 8 h of stimulation and then declined but remained elevated after 24 h of stimulation (Figure 1A). Cells treated with 5 μg/mL of visfatin displayed the maximal induced level of COX2 expression compared to those treated with lower concentrations (Figure 1B). As the inactivation of GSK3β has been implicated in both cartilage development and the pathogenesis of OA [26,27], we further determined if GSK3β activity was modulated in this effect of visfatin on human primary chondrocytes. Cells were kept as controls or treated with 5 μg/mL of visfatin for 1, 4, 8 and 24 h, and then GSK3β activity was determined by analyzing its Serine-9 residue phosphorylation, which was indicated as GSK3β inactivation. Treatment of human primary chondrocytes with visfatin induced GSK3β-Ser9 phosphorylation within 1 h and persisted for 24 h (Figure 1C). Cells were pretreated with DMSO or DIF-3, a GSK3β activator, for 1 h and then kept as controls or treated with 5 μg/mL of visfatin for 8 h. COX2 expression levels in human primary chondrocytes were analyzed. It was observed that pretreating cells with DIF-3 significantly decreases the COX2 expression levels in human primary chondrocytes in response to visfatin (Figure 1D).

### 2.2. P38 Signaling Regulates the Effect of Visfatin on GSK3β Inactivation and Subsequent COX2 Expression in Human Primary Chondrocytes

**Figure 2:**
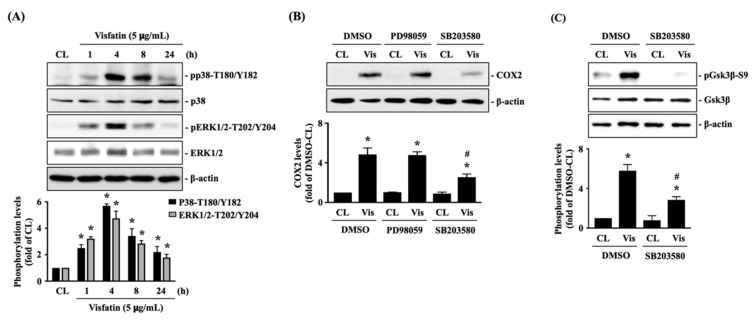
p38 signaling regulates the effect of visfatin on GSK3β inactivation and subsequent COX2 expression in human primary chondrocytes. (**A**) Human primary chondrocytes were kept as controls (CL) or treated with 5 μg/mL of visfatin for 1, 4, 8 and 24 h, and then the p38-Thr180/Tyr182 and ERK1/2-Thr202/Tyr204 kinases phosphorylations were analyzed by Western blot. (**B**,**C**) Human primary chondrocytes were pretreated with DMSO, PD98059 (ERK1/2 inhibitor, 30 μM), or SB203580 (p38 inhibitor, 10 μM) for 1 h and then kept as controls (CL) or treated with 5 μg/mL of visfatin (Vis) for 8 h. COX2 expression (**B**) and GSK3β-Ser-9 phosphorylation (**C**) in human primary chondrocytes were analyzed by Western blot. Results in (**A**–**C**) are representative of three independent experiments with similar results. Statistic data in (**A**–**C**) are mean ± SEM from three independent experiments. *, *p* < 0.05 vs. untreated control cells (CL) or DMSO-CL-treated cells. #, *p* < 0.05 vs. DMSO-visfatin (Vis)-stimulated cells.

Human primary chondrocytes were kept as controls or treated with 5 μg/mL of visfatin for 1, 4, 8 and 24 h, and then the phosphorylations of p38-Thr180/Tyr182 (activation) and ERK1/2-Thr202/Tyr204 (activation) kinases were analyzed. Phosphorylation was induced in both p38 and ERK1/2 kinases in cells treated with visfatin within 1 h. The maximal inductions of p38 and ERK1/2 kinases phosphorylations appeared after 4 h of stimulation and then declined but remained elevated after 8 h and 24 h of stimulations (Figure 2A). Cells were pretreated with DMSO, PD98059 (ERK1/2 inhibitor, 30 μM), or SB203580 (p38 inhibitor, 10 μM) for 1 h and then kept as controls or treated with 5 μg/mL of visfatin for 8 h. COX2 expression levels in human primary chondrocytes were analyzed. It was shown that pretreating cells with SB203580, but not PD98059, significantly decreased the COX2 expression levels in cells in response to visfatin (Figure 2B). Moreover, SB203580 pretreatment also blocked the visfatin-induced GSK3β-Ser9 phosphorylation (GSK3β inactivation) in human primary chondrocytes (Figure 2C).

### 2.3. Visfatin Changes the Intracellular Mechanics of Human Primary Chondrocytes 

**Figure 3:**
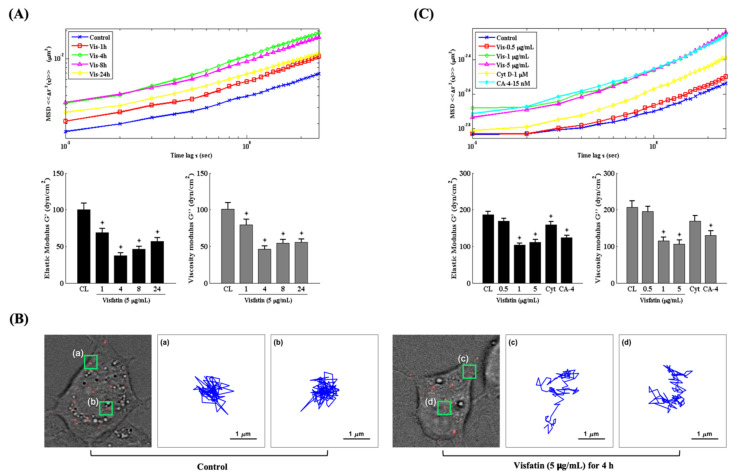
Visfatin changes the intracellular mechanics of human primary chondrocytes. (**A**–**C**) Human primary chondrocytes were injected with fluorescent beads and then kept as controls (CL) or treated with 5 μg/mL of visfatin for 1, 4, 8 and 24 h or treated with 0.5, 1 or 5 μg/mL of visfatin for 4 h. (**A**,**C**) The intracellular mechanics, i.e., the MSD and elasticity and viscosity moduli of human primary chondrocytes were analyzed by using the PTM method. (**B**) Actual examples of intracellular particle-tracking microrheology and the Brownian motion trajectory of the selected fluorescent beads at untreated control cells (a and b, left three panels) and 5 μg/mL of visfatin treatment for 4 h (c and d, right three panels). *, *p* < 0.05 vs. untreated control cells (CL).

Changes in the intracellular mechanics of chondrocytes have been implicated in modulating cartilage homeostasis and OA pathogenesis [16,22,28]. Therefore, we explored if the catabolic effects of visfatin affect the intracellular mechanics of human primary chondrocytes. Cells were injected with fluorescent beads and then kept as controls or treated with 5 μg/mL of visfatin for 1, 4, 8 and 24 h or treated with 0.5, 1 or 5 μg/mL of visfatin for 4 h. The intracellular mechanics of human primary chondrocytes were analyzed by using the PTM method [25]. It was observed that 5 μg/mL of visfatin decreased the intracellular elasticity and viscosity within 1 h in the cells, which reached a maximal effect after 4 h of stimulation and then gradually recovered but remained effective after 24 h of stimulation (down panel in Figure 3A). The MSD trajectory analysis showed that the beads in visfatin-treated cells had a higher microrheology property and displayed more random displacement compared to untreated cells (up panel in Figure 3A,B). Cells treated with 1 μg/mL and 5 μg/mL (not 0.5 μg/mL) of visfatin showed similar results in regulating the intracellular mechanics (Figure 3C). Cells treated with cytochalasin D (Cyt D, 1 μM), an actin destabilizer, partially affected the intracellular mechanics of human primary chondrocytes, but cells treated with combretastatin-4 (CA-4, 15 nM), a microtubule destabilizer, elicited a significant response at an efficiency similar to the 1 and 5 μg/mL visfatin-treated cells (Figure 3C). 

### 2.4. Visfatin Destabilizes the Microtubule Network to Influence Its Catabolic Effect on COX2 Expression in Human Primary Chondrocytes

**Figure 4:**
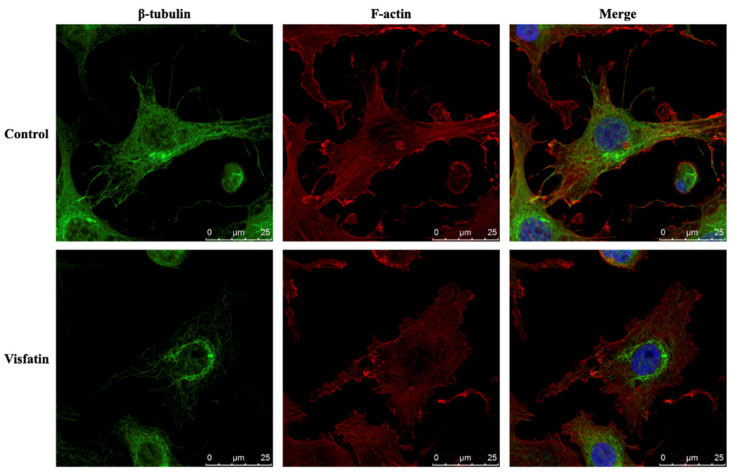
Visfatin destabilizes microfilament and microtubule networks in human primary chondrocytes. Human primary chondrocytes were kept as controls or treated with 5 μg/mL of visfatin for 4 h. The microfilament and microtubule networks were stained with Alexa Fluor 633-phalloidin (F-actin, red color) and β-tubulin-specific antibody with Alexa Fluor 488-2nd antibody (green color), respectively. The nucleus was stained with DAPI. The fluorescent images were acquired on Leica TCS SP5 II confocal microscope. The scale bar is 25 μm with 5 μm intervals.

**Figure 5:**
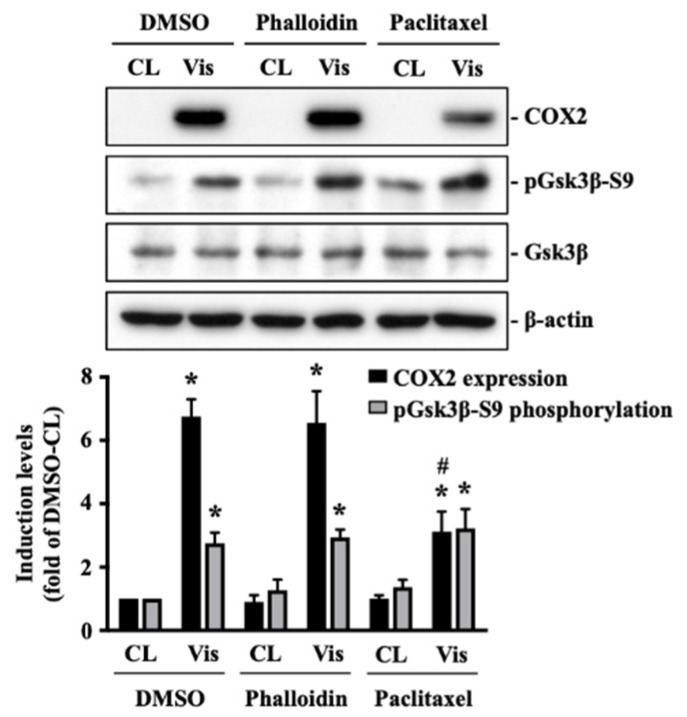
Microtubule network damage influences the visfatin catabolic effect on COX2 upregulation in human primary chondrocytes. Human primary chondrocytes were pretreated with DMSO, phalloidin (microfilament stabilizer, 3 μM), or paclitaxel (microtubule stabilizer, 1 μM) for 1 h and then kept as controls (CL) or treated with 5 μg/mL of visfatin (Vis) for 8 h. COX2 expression and GSK3β-Ser9 phosphorylation in human primary chondrocytes were analyzed by Western blot. Results are representative of three independent experiments with similar results. Statistic data are mean ± SEM from three independent experiments. *, *p* < 0.05 vs. DMSO-CL-treated cells. #, *p* < 0.05 vs. DMSO-visfatin (Vis)-stimulated cells.

The polymerization status of cytoskeletal architecture has been indicated as the major controller of the intracellular mechanics of cells [20,29]. Therefore, we further determined if visfatin affects the microfilament and microtubule networks in human primary chondrocytes. Cells were kept as controls or treated with 5 μg/mL of visfatin for 4 h and then the F-actin and β-tubulin fiber networks were analyzed. Treating cells with visfatin significantly decreased the β-tubulin and F-actin fibers in human primary chondrocytes (Figure 4). Cells were pretreated with DMSO, phalloidin (microfilament stabilizer, 3 μM), or paclitaxel (microtubule stabilizer, 1 μM) for 1 h and then kept as controls or treated with 5 μg/mL of visfatin for 8 h. COX2 expression and GSK3β-Ser9 phosphorylation levels in human primary chondrocytes were analyzed. It was shown that pretreating cells with paclitaxel, but not phalloidin, significantly decreased the COX2 expression levels in human primary chondrocytes in response to visfatin (Figure 5). However, both the paclitaxel and phalloidin pretreatment did not affect the visfatin-induced GSK3β-Ser9 phosphorylation (GSK3β inactivation) in human primary chondrocytes (Figure 5).

### 2.5. GSK3β Inactivation Regulates the Visfatin-Induced Changes in Intracellular Mechanics and Cytoskeletal Architecture in Human Primary Chondrocytes

**Figure 6:**
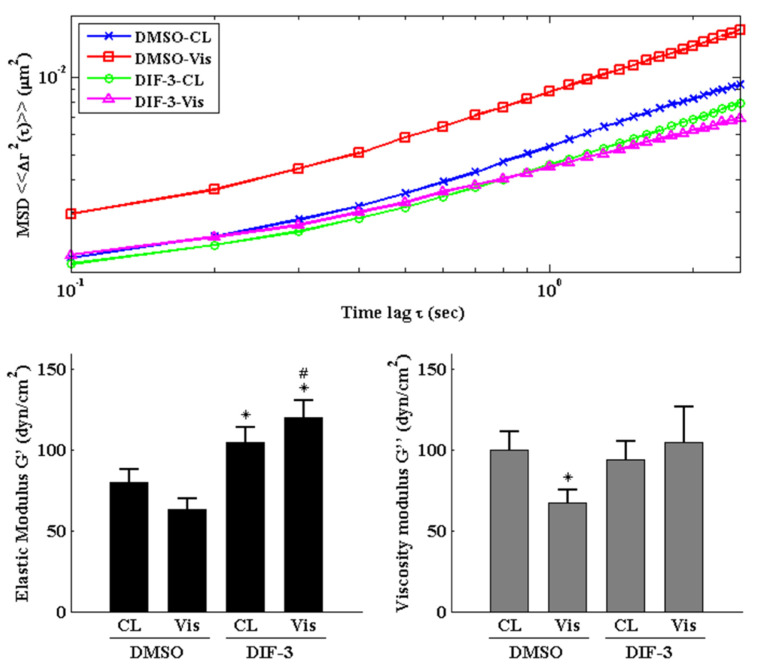
GSK3β inactivation regulates visfatin-induced changes in intracellular mechanics in human primary chondrocytes. Human primary chondrocytes were pretreated with DMSO or DIF-3, a GSK3β activator, for 1 h and then kept as controls (CL) or treated with 5 μg/mL of visfatin (Vis) for 4 h. The intracellular mechanics, i.e., the MSD and elasticity and viscosity moduli, of human primary chondrocytes were analyzed using the PTM method. *, *p* < 0.05 vs. DMSO-CL-treated cells. #, *p* < 0.05 vs. DMSO-visfatin (Vis)-stimulated cells.

**Figure 7:**
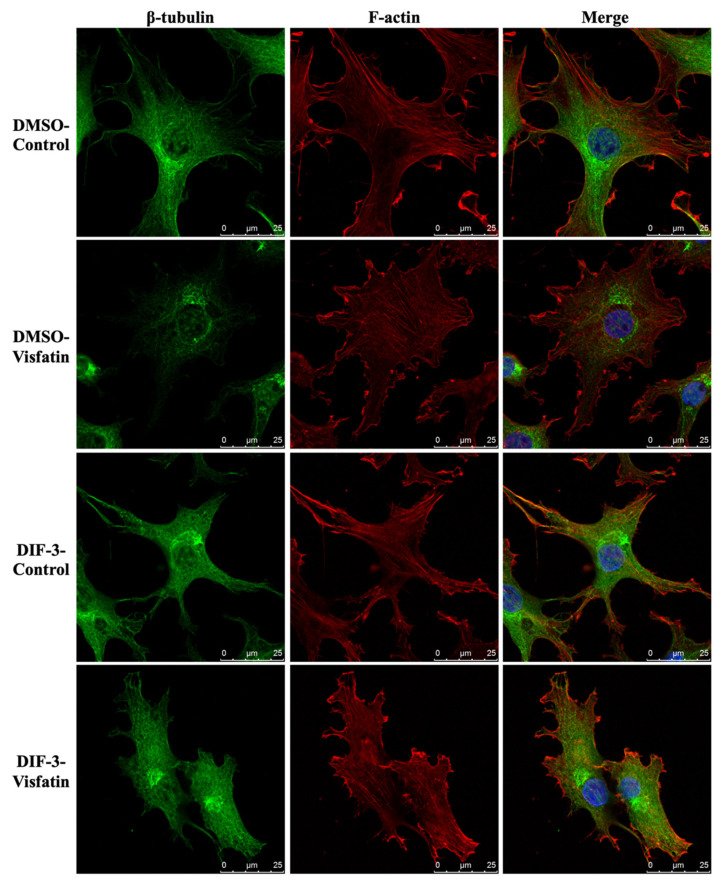
GSK3β inactivation regulates visfatin-induced damage of the microfilament and microtubule networks in human primary chondrocytes. Human primary chondrocytes were pretreated with DMSO or DIF-3, a GSK3β activator, for 1 h and then kept as controls (CL) or treated with 5 μg/mL of visfatin (Vis) for 4 h. The microfilament and microtubule networks were stained with Alexa Fluor 633-phalloidin (F-actin, red color) and β-tubulin-specific antibody with Alexa Fluor 488-2nd antibody (green color), respectively. The nucleus was stained with DAPI. The fluorescent images were acquired on Leica TCS SP5 II confocal microscope. The scale bar is 25 μm with 5 μm intervals.

Cells were pretreated with DMSO or DIF-3, a GSK3β activator, for 1 h and then kept as controls or treated with 5 μg/mL of visfatin for 4 h. The intracellular mechanics along with F-actin and β-tubulin fiber networks in these cells were analyzed. It was revealed that pretreating cells with DIF-3 recovered the visfatin effects on decreasing the elasticity and viscosity properties at different levels and increasing the random displacement property of beads in human primary chondrocytes (Figure 6). Moreover, DIF-3 pretreatment also recovered the lowered levels of β-tubulin and F-actin fibers in human primary chondrocytes induced by visfatin (Figure 7).

## 3. Discussion

**Figure 8:**
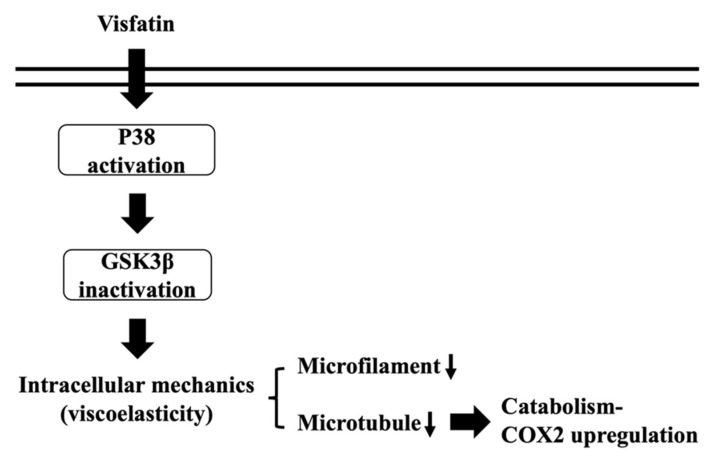
Schematic representation of the signaling regulating visfatin-induced effects on intracellular mechanics and catabolism in human chondrocytes.

Because of remarkable advances of new techniques and methods, accumulating data have revealed the role and importance of intracellular biophysical behavior in controlling cell physiology and pathophysiology. Chondrocytes have been indicated as one of the most impacted cell types because cartilage and its chondrocytes are constantly subjected to internal and external mechanical stress. Moreover, these mechanical controls have been implicated in maintaining metabolic homeostasis or promoting OA, the underlying mechanisms of which still needs more understanding. Obesity is still the most predominant risk factor of OA because of weight overload and simultaneous adipokine inducing effects. Recently, the involvement of visfatin in OA became relevant because of the clinical findings of the positive association between the serum and synovial levels of this molecule and OA progression. Accordingly, the systematic experiments in the present study demonstrated that (i) the change in intracellular mechanics in human primary chondrocytes occurs prior to the induction of catabolism upon visfatin stimulation; (ii) GSK3β inactivation induced by p38 signaling is responsible for both the intracellular mechanics and catabolism regulation caused by visfatin in human primary chondrocytes; (iii) damage to both microfilament and microtubule networks account for the visfatin-induced changes in intracellular mechanics; (iv) damage to the microtubule network, but not the microfilament network, affects the catabolic effect of visfatin on COX2 upregulation (summarized in Figure 8).

Our results found that t catabolic COX2 upregulation in visfatin-stimulated human primary chondrocytes occurs following the change in intracellular mechanics, i.e., a softer cytoplasmic environment due to decreased elasticity and viscosity. Moreover, the time-course change of the intracellular mechanics of human primary chondrocytes was similar to that of COX2 upregulation via visfatin induction. Previous studies have suggested that the mechanical property of the cartilage matrix may show a shift from stiffness to softness due to a faster rate of catabolism than anabolism during the processes of inflammation and degeneration [30,31]. In that case, the surrounding mechanical microenvironment of chondrocytes is also altered, making the dynamic regulation of intracellular mechanics necessary for the cells to change their phenotype to adapt to unusual mechanical loading and pressure [31]. Interestingly, although our finding indicated that visfatin-treated chondrocytes had decreased intracellular mechanics, which was supported by some other studies, other research team reported the opposite observation, i.e., that the chondrocytes in OA are stiffer than the healthy ones [16,17,18,22]. Possible interpretations for the changes in intracellular mechanics and those different observations have been proposed as follows: (i) chondrocytes become stiffer or softer in accordance with the complex stress in the environment; (ii) chondrocytes must reactivate themselves to synthesize more anabolic factors to repair damage; (iii) chondrocytes mostly communicate with a weak and low-strength matrix [31]. In any case, the changes in intracellular mechanics have received much attention. Interest concerning on the causes behind the changes in different situations, and interactions with the biochemical signaling regulation, is increasing.

The major functions of the cytoskeleton in cells are to maintain the cell morphology and form the communication channel between the intracellular cytoplasm and the extracellular matrix [20]. In the present study, we found that microfilament and microtubule networks are damaged, with visfatin decreasing the intracellular mechanics of chondrocytes. Moreover, the destruction of the microtubule network further affected the catabolic effect of visfatin on COX2 upregulation. In the cartilage, although three types of cytoskeletal networks have all been indicated as controlling the intracellular mechanics, the microfilament network has been recognized as the predominant one [20]. Many previous studies also demonstrated the importance of the microfilament network in regulating the viscoelasticity and stiffness properties of chondrocytes, including cells treated with interleukin-1β-/tumor necrosis factor α and cytoskeleton destabilizer agents [17,18,22]. However, the microtubule network was found to have little effect on the biophysical behaviors of chondrocytes in these studies [17,18,22]. These findings are different from ours. However, earlier work on endothelial cells, neutrophils, and hepatocytes showed the effect of the microtubule network on the regulation of intracellular mechanics in these cells [32,33,34]. It was suggested that the difference might result from the various stimuli and/or the cell status, e.g., cell division. Moreover, apart from accounting for cell shape, another function of the microtubule network is to control the intracellular polarity of the cells, which might also change intracellular mechanics [22]. Thus, the correlations between cell status (including polarity), cell mechanics and catabolism in chondrocytes in response to visfatin still warrant further investigation. 

Our results further showed that GSK3β inactivation might be a pivotal trigger of the catabolic effect of visfatin on damage to microfilament and microtubule networks, and COX2 upregulation. GSK3β activity controlled by phosphorylation status via various stimuli with downstream kinases is important in regulating many cellular functions and is concerned in development and therapy of many diseases [35]. In the cartilage, cell fate decision by GSK3β activity is also dependent on the context. For example, during endochondral ossification, GSK3β inactivation by cGMP-dependent protein kinase II promotes hypertrophy and terminal differentiation of chondrocytes [36], while GSK3β inactivation-activated β-catenin signaling promotes the chondrogenesis of bone marrow stem cells by rearranging the microfilament network upon stimulation by vibration [37]. GSK3β activity has also been suggested to serve as a checkpoint to maintain the mature chondrocytes in a healthy metabolic homeostasis status by finely tuning the overwork of β-catenin signaling [38,39]. Moreover, accumulating data has shown a higher level of GSK3β inactivation in the cartilage of obese OA patients, and that such GSK3β inactivation could lead to mitochondrial dysfunction, matrix remodeling and, finally, OA development [26,27]. Combining all these points, it appears that the GSK3β activity in chondrocytes/cartilage is dependent on the differentiation, maturation timing and stage of the cells. The optimal maintenance of GSK3β activity in healthy cartilage is necessary and, therefore, GSK3β inactivation might serve as a potential theranostic target for OA therapy.

The limitations of the present work should be noted. First, the primary chondrocytes used in this study were all isolated from OA patients undergoing joint replacement because the collection of non-OA and non-diseased cartilage tissues, e.g., from a patient with severe trauma, is difficult. This also means that the OA status of most patients was at a late or severe stage. We did not further examine if chondrocytes from OA patients with different severity might affect their response to visfatin in this work. However, collagen II/aggrecan expression levels in the isolated primary chondrocytes were examined to check the physiological condition. Second, accumulating evidence has elucidated the importance of intracellular mechanics in regulating physiology and pathophysiology, but most studies, including ours, could use only cell models to perform the investigation. It is still too early, and difficult, to examine this topic through in vivo design.

## 4. Materials and Methods

### 4.1. Materials

Recombinant human visfatin (130-09) was obtained from PeproTech (Rocky Hill, NJ, USA). COX2-specific antibody (PA5-17614) was obtained from Thermo (Waltham, MA, USA). Specific antibodies for pGSK3β-Ser9 (#9336), GSK3β (#9315), pp38-Thr180/Tyr182 (#4511), p38 (#9212), pERK1/2-Thr202/Tyr204 (#4370), ERK1/2 (#4695), and β-actin (#3700) were obtained from Cell Signaling Technology (Beverly, MA, USA). Alexa Fluor 633-phalloidin (A22284) and DAPI (D1306) were obtained from Invitrogen (Carlsbad, CA, USA). β-tubulin-specific antibody (ab6045) and 2nd antibody-Alexa Fluor 488 (ab150077) were obtained from Abcam (Cambridge, MA, USA). DIF-3 (D0567), PD98059 (P215), SB203580 (S8307), cytochalasin (C82723), combretastatin A4 (C7744), phalloidin (P2141), and paclitaxel (T7402) were obtained from Sigma (St. Louis, MO, USA). All other chemicals were obtained from Sigma (St Louis, MO, USA). 

### 4.2. Cell Culture

Human primary chondrocytes used in all the experiments were isolated from OA patients who underwent knee surgery. The OA patients’ clinical diagnosis depended on the American college of rheumatology criteria for OA knee [40]. The Ethics Committees in Institutional Review Board of Chang Gung Memorial Hospital reviewed and approved the study method and protocol (IRB: 201602021B0, 1 August 2017~31 July 2021). We have got the written informed consents from the patients. Primary chondrocyte isolation was conducted according to our published papers [41]. In brief, fresh and healthy regions of the patient’s cartilage were sliced into pieces (~0.5 cm^2^). These pieces were further digested with 2 mg/mL of pronase/0.25 mg/mL of collagenase I (Calbiochem, La Jolla, CA, USA) and then the solution, including chondrocytes, was filtered through a 100 μm-pore size mesh. The isolated primary cells were cultured in DMEM medium (Sigma, St Louis, MO, USA) with 10% fetal bovine serum (Thermo, Waltham, MA, USA) and 1% antibiotics (penicillin and streptomycin) (Thermo, Waltham, MA, USA) until confluence. In our experiments, only 1st-passage chondrocytes were used.

### 4.3. Western Blot

Total proteins were collected from human primary chondrocytes lysed with commercial lysis buffer (20-188, Millipore, Darmstadt, Germany) adding a protease/phosphatase inhibitor cocktail (04906837001, Roche, Basel, Switzerland). Total cell lysates were quantified by the Bradford method and 30 µg of protein lysates were separated by SDS-PAGE and then transferred onto 0.45 µm-pore size nitrocellulose paper (Bio-Rad, Richmond, CA, USA). Specific protein analysis on the papers was conducted by adding 1st antibodies and 2nd antibodies conjugated with horseradish peroxidase and analyzed in a Western-Light chemiluminescent detection system (Applied Biosystems, Foster, CA, USA). The signal intensities of the bands were analyzed using ImageJ software.

### 4.4. Injection of Fluorescent Beads

Fluorescent beads (0.1 µm; F8801, Invitrogen, Carlsbad, CA, USA) were injected into cells using the PDS-1000/He biolistic particle delivery system (Bio-Rad, Richmond, CA, USA) with 450 psi-pressure. Images of fluorescence beads were acquired on a Leica TCS SP5 II confocal microscope (Leica, Solms, Germany) equipped with a 63×/1.4NA HCX PL APO oil objective with an additional 3× zoom. The signal was excited by a DPSS 561 nm laser with minimized light intensity settings and exposure time to preclude causing phototoxicity. Adaptive Focus Control and a Chamlide top-stage incubator system were used to avoid focal drift and keep the cells in steady situations. The sampling rate of the 10-s time lapse images was 10 frames/sec with a resolution of 160.5 nm/pixel and 512 × 195 pixels per frame. 

### 4.5. Tracking of Fluorescent Beads

The images of the fluorescence beads with Brownian motion were analyzed using our home-made MATLAB programs. The position of a selected bead in an image frame was set to be the mean of the pixels’ positions weighted by each pixel’s intensity (i.e., the weighted center of mass) of the image pixels the bead covered [42]. To link the beads in a sequence of frames, we assumed that the closest beads in two nearby frames were the same [43]. Given the positions of a selected bead in these frames, the two-dimensional trajectory over time could be derived, and the mean-squared displacements (MSDs), $\langle \Delta r^{2}\left(\tau \right)\rangle$, were calculated using

(1) $$\langle \Delta r^{2}\left(\tau \right)\rangle =\langle {\left[x\left(t+\tau \right)-x\left(t\right)\right]}^{2}+{\left[y\left(t+\tau \right)-y\left(t\right)\right]}^{2}\rangle$$

where *x*(*t*) and *y*(*t*) are the bead positions at the time *t*, and *τ* is the time lag. MSDs grow as a function of time and can be described with a power law, $\langle \Delta r^{2}\left(\tau \right)\rangle \sim \tau^{\alpha }$, when beads diffuse in viscoelastic media. The exponent α is the slope of the log-log plot of the MSDs, called the diffusive exponent. Subdiffusive behavior inside a complex medium is described by an α with a value lower than one. If α>1, active transport driven by forces other than thermal fluctuations may be involved and the deduction of viscoelastic moduli are no longer valid [43,44,45]. The bead trajectories exhibiting non-Brownian motion (i.e., those with slopes more than one) were discarded before the calculation of the viscoelastic modulus:

(2) $$G^{*}\left(\omega \right)=\frac{k_{B}T}{\pi ai\omega F_{u}\left[\langle \Delta r^{2}\left(\tau \right)\rangle \right]}$$

where $F_{u}\left[\langle \Delta r^{2}\left(\tau \right)\rangle \right]$ is the unilateral Fourier transform of $\langle \Delta r^{2}\left(\tau \right)\rangle$, *k*_B_ is the Boltzmann constant, *T* is the absolute temperature, and *a* is the radius of the bead. Allowing the transform to be estimated algebraically by expanding $\langle \Delta r^{2}\left(\tau \right)\rangle$ locally around the frequency ω of interest using a power law, we have

(3) $$G^{*}\left(\omega \right)\approx \frac{k_{B}T}{\pi a\langle \Delta r^{2}\left(\frac{1}{\omega }\right)\rangle \Gamma \left[1+\alpha \left(\omega \right)\right]}e^{i\frac{\pi \beta \left(\omega \right)}{2}}$$

with Γ being the gamma function and $\beta \left(\omega \right)={\frac{dln\langle \Delta r^{2}\left(\tau \right)\rangle }{dln\tau }|}_{\tau =1/\omega }$. Finally, by applying Euler’s formula, (3) can be rewritten as $G^{*}\left(\omega \right)=G^{\prime }\left(\omega \right)+iG^{\prime \prime }\left(\omega \right)$ with $G^{\prime }\left(\omega \right)$ and $G^{\prime \prime }\left(\omega \right)$ being the elastic modulus and the viscous modulus, respectively. As in several existing studies [25], the elastic and viscous moduli were evaluated at 1 Hz for a given ensemble-averaged MSD curve. Finally, the statistical significance was computed by the Mann-Whitney U test.

### 4.6. Immunofluorescent Staining

Human primary chondrocytes were fixed with 4% paraformaldehyde and permeabilized with 0.25% Triton X-100. Cells were blocked with 1% bovine serum albumin and then the microfilament and microtubule networks were stained with Alexa Fluor 633-phalloidin (F-actin) and β-tubulin-specific antibody with Alexa Fluor 488-2nd antibody. Fluorescent images were acquired on Leica TCS SP5 II confocal microscope.

### 4.7. Statistical Analysis

Results were specified and analyzed by mean ± SE computed by the independent Student t-test for two groups of data and ANOVA, followed by Scheffe’s test for multiple comparisons. Significance was set at *p* < 0.05. All the experiments were repeated three-times from individual samples.

## 5. Conclusions 

In summary, our findings indicate that visfatin can destroy microtubule and microfilament networks to affect the intracellular mechanics of human primary chondrocytes via GSK3β inactivation induced by p38 signaling. Moreover, microtubule network destruction in human primary chondrocytes is predominantly responsible for the catabolic effect of visfatin by COX2 upregulation. With advances in new techniques, accumulating data show the importance of regulating intracellular mechanics in disease progression. Our study shows a more comprehensive interpretation of OA development from biochemical and biophysical perspectives. Further, GSK3β inactivation, and subsequent regulation of intracellular mechanics, might be considered a theranostic target for future medication development for OA.

## Data Availability

The data that support the findings of current study are available from the corresponding author upon reasonable request.
